# Deep Layer Kernel Sparse Representation Network for the Detection of Heart Valve Ailments from the Time-Frequency Representation of PCG Recordings

**DOI:** 10.1155/2020/8843963

**Published:** 2020-12-21

**Authors:** Samit Kumar Ghosh, R. N. Ponnalagu, R. K. Tripathy, U. Rajendra Acharya

**Affiliations:** ^1^Department of Electrical and Electronics Engineering, BITS-Pilani, Hyderabad Campus, Hyderabad 500078, India; ^2^Department of Electronics and Computer Engineering, Ngee Ann Polytechnic, Singapore

## Abstract

The heart valve ailments (HVAs) are due to the defects in the valves of the heart and if untreated may cause heart failure, clots, and even sudden cardiac death. Automated early detection of HVAs is necessary in the hospitals for proper diagnosis of pathological cases, to provide timely treatment, and to reduce the mortality rate. The heart valve abnormalities will alter the heart sound and murmurs which can be faithfully captured by phonocardiogram (PCG) recordings. In this paper, a time-frequency based deep layer kernel sparse representation network (DLKSRN) is proposed for the detection of various HVAs using PCG signals. Spline kernel-based Chirplet transform (SCT) is used to evaluate the time-frequency representation of PCG recording, and the features like L1-norm (LN), sample entropy (SEN), and permutation entropy (PEN) are extracted from the different frequency components of the time-frequency representation of PCG recording. The DLKSRN formulated using the hidden layers of extreme learning machine- (ELM-) autoencoders and kernel sparse representation (KSR) is used for the classification of PCG recordings as normal, and pathology cases such as mitral valve prolapse (MVP), mitral regurgitation (MR), aortic stenosis (AS), and mitral stenosis (MS). The proposed approach has been evaluated using PCG recordings from both public and private databases, and the results demonstrated that an average sensitivity of 100%, 97.51%, 99.00%, 98.72%, and 99.13% are obtained for normal, MVP, MR, AS, and MS cases using the hold-out cross-validation (CV) method. The proposed approach is applicable for the Internet of Things- (IoT-) driven smart healthcare system for the accurate detection of HVAs.

## 1. Introduction

The heart valve ailments (HVAs) are cardiovascular abnormalities, and these ailments occur due to the defect in any of the valves (tricuspid, pulmonary, mitral, and aortic) of the heart [1, 2]. The valves of the heart prevent the backward flow of the blood, and for the proper functioning of the heart, the valve should be effectively closed or opened during the flow of blood from one chamber to another chamber of the heart [3]. The HVAs are classified as mitral stenosis (MS), mitral valve prolapse (MVP), mitral regurgitation (MR), and aortic stenosis (AS) based on the defect in the heart valves [4]. The MR ailments occur due to the improper closing of the mitral valve, which further causes the reverse flow of blood from the left ventricle to the left atrium [5]. Similarly, the AR refers to the improper closing of the aortic valve; as a result, the backward flow of blood from the aorta to the right ventricle may occur [5]. Moreover, the MS is termed as the problem in the opening of the mitral valve, where the left ventricle is not getting a sufficient amount of blood from the left atrium [6]. Similarly, the AS pathology refers to the improper opening of the aortic valve, which prevents the flow of blood from the left ventricle to the aorta of the heart [5] [6]. For the diagnosis of these pathologies, different imaging techniques such as computed tomography scan, magnetic resonance imaging (MRI), cardiac echocardiography, and ultrasonic devices have been used [7–10]. It has been reported from the literature that various quantitative parameters such as transvalvular velocity, average value area, and mean value of transvalvular gradient have been considered to determine the progression of HVAs [11]. The aforementioned imaging modalities have limitations, such as the selection of tuning parameters in ultrasonic devices to obtain better resolution images of heart chambers and valves for the diagnosis of HVAs [10, 12]. Also, these imaging techniques are costly and require trained medical staff for the accurate assessment of HVAs [13]. The phonocardiography (PCG) is a noninvasive and low-cost diagnostic test used for the detection of HVAs [14, 15]. The diagnostic features such as the duration of both the systolic segment and diastolic segment, morphologies of both S1 and S2 components, and the appearance of murmurs have been investigated for the diagnosis of HVAs [14, 16]. To assist the clinicians in the diagnosis of HVAs, an automatic diagnosis system (ADS) will be helpful especially while treating patients admitted in the intensive care unit where continuous recording and monitoring of PCG signal is done 24 hours [17]. The ADS comprises the evaluation of various diagnostic features from the PCG recording and automated classification of HVAs using the PCG signal features [13]. For smart healthcare and the Internet of healthcare things (IoHT) applications [18, 19], the automated diagnosis of HVAs from the PCG signal is a challenging area of research. Therefore, the development of new methods for the extraction of PCG signal features and the classification of HVAs is required.

In the last two decades, various algorithms have been used for the automated detection of HVAs using PCG signals. These algorithms have considered different feature extraction methodologies to extract the features from the PCG signal and used various machine learning classifiers for the categorization of HVAs. A review of various automated methods for the detection of HVAs has been reported in [20, 21]. The time, frequency, time scale, and time-frequency domain-based features from PCG signal have been used for the detection of HVAs. The time-domain features from the PCG signals have been used in [22–26], for the categorization of both normal and abnormal heart sounds. Similarly, in [27–30], the frequency domain features from the PCG signals have been considered for the discrimination of normal and abnormal cardiac sounds. The time-scale-based methods such as discrete wavelet transform (DWT) [31, 32], empirical mode decomposition (EMD) [31, 32], and tunable Q-wavelet transforms (TQWT) [33] of PCG signals have also been used for the detection of HVAs. Moreover, the time-frequency analysis-based approaches such as the short-time Fourier transform (STFT) [34, 35], synchrosqueezing transform (SST) [36], and other time-frequency decomposition-based approaches [37–39] of PCG signals are used for the categorization of HVAs. The machine learning techniques such as the support vector machines (SVM) [40], random forest (RF) [41], convolutional neural network (CNN) [42], and hidden Markov model (HMM) [43] have been used for the classification of HVAs. It is evident from the literature that time-frequency and time-scale analysis-based approaches have demonstrated higher classification performance for the detection of HVAs using PCG signals. Son et al. [44] have combined the Mel frequency cepstral coefficients (MFCC) and DWT-based features from the PCG signals and used these features for the detection of HVAs. They have considered various machine learning classifiers for HVA detection. In [45], the authors have applied a novel algorithm based on wavelet fractal dimension and a twin support vector machine (TWSVM) for the classification of HVAs using PCG signals. Moreover, Ghosh et al. [36] have extracted the magnitude and phase features from the time-frequency representation of the segmented PCG cycles for the discrimination of HVAs. They have used synchrosqueezing transform (SST) for the evaluation of the time-frequency matrix from the PCG signal. The SST-based method has drawbacks such as it has poor time-frequency resolution for PCG signals as it uses the coefficient reassignment in the time-frequency plane based on the instantaneous frequency of the PCG signal [36, 46]. Also, the SST method has shown less performance for the detection of HVAs. The methods reported in the literature have segmented the PCG signal into cardiac cycles and then extracted features from the segmented cardiac heart sound cycles for the detection of HVAs. The PCG signal with multiple cardiac heart sound cycles effectively captures the variations in the amplitudes and shapes of S1 and S2 sound components and the duration of systolic and diastolic segments [14]. The existing approaches have not considered the PCG signals from all HVA classes to design the automated diagnosis frameworks. Therefore, an intelligent system which uses PCG signal with multiple cardiac heart sound cycles and classifies all HVAs is required for healthcare applications.

The PCG signal is nonstationary, and the components of this signal such as S1, S2, and murmurs are nonlinear and time-varying [47, 48]. In our previous work, we have analyzed the PCG signal using Chirplet transform (CT) for the detection of HVAs [44]. The CT works well for chirp-like signals with linearly time-varying components [49, 50]. But the CT fails to capture the transition from S1 component to systolic murmur, and from S2 component diastolic murmur in the time-frequency plot of the pathological PCG signals [13]. In this work, we have considered the spline CT (SCT) as the extension of CT for the evaluation of the time-frequency matrix from the PCG signal. The SCT has advantages such as it has better time-frequency localization for the nonlinearly time-varying components of the nonstationary signal as compared to CT [51]. Therefore, we can expect that the time-frequency matrix computed using SCT of the PCG signal can effectively capture the pathological variations and provide better resolution in the time-frequency domain of the PCG signal. Recently, the convolutional neural network (CNN) and stacked autoencoder- (SAE-) based deep neural network (DNN) methods have been used for the automated assessment of HVAs using PCG signals [44, 52]. In order to obtain the optimal parameters in CNN and SAE networks, rigorous training based on the gradient descent algorithm is used [53]. Also, these networks require more instances during the training process for obtaining the optimal model parameters [54]. The DNN based on extreme learning machine- (ELM-) autoencoder has advantages such as it requires less training time for the evaluation of the model parameters [55], and the ELM-autoencoder model can be efficiently implemented in real-time for the dimension reduction [56]. The sparse representation-driven classification methods have been widely used for various biomedical applications [13, 57–59]. These methods require fewer features for training instances and also have fewer training parameters for the prediction of class labels from the test feature vectors [59]. The SRC has shown better performances as compared to other machine learning approaches for the classification of HVAs from PCG signal features [13]. The kernel sparse representation classifier (KSRC) uses the kernel trick to map the feature instances to the higher dimensional space, and the SRC is applied in the higher dimensional space for the classification [60, 61]. The KSRC has shown better classification performance for the dataset which consists of nonlinearly separable feature instances as compared to SRC [57, 62]. Therefore, the DNN developed based on the ELM-autoencoder, and KSRC will be effective for the automated detection of HVAs using the time-frequency representation of the PCG signal. The contributions of this paper are written as follows:

- The SCT-based time-frequency analysis is used for the evaluation of time-frequency representation of PCG recording
- The nonlinear features such as the L1-norm (LN), sample entropy (SEN), and permutation entropy (PEN) are computed from different frequency components of the SCT-based time-frequency matrix of PCG signals
- The deep layer kernel sparse representation network (DLKSRN) is proposed for the detection of HVAs using the time-frequency domain features of the PCG signal

The remaining sections of this manuscript are written as follows. In Section 2, the proposed method for the detection and classification of HVAs is described. The results obtained from the proposed work are discussed in Section 3, and conclusions are presented in section 4.

## 2. Proposed Method

**Figure 1:**
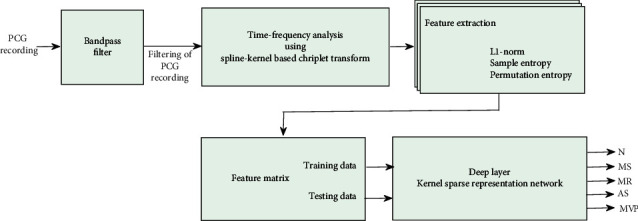
Flow diagram of the proposed approach for HVAs detection.

The flow diagram of the proposed HVA detection approach is depicted in Figure 1, and the details of the various steps involved in the proposed approach are explained in detail in the following subsection.

### 2.1. PCG Signal Collection and Filtering

In this work, we have collected the PCG recordings from a public database available in (https://github.com/yaseen21khan/Classification-of-Heart-Sound-Signal-Using-Multiple-Features-). The detailed description of the PCG signals database is given in [44]. The database contains a total of 1000 PCG recordings of different classes. Out of those 1000 recordings, each class (normal or pathological) contains 200 PCG recordings. The annotations for the PCG signals for normal (N) and pathological (MS, MR, AS, and MVP) classes are given in the database. The PCG recordings are given in wav file format, and these signals were recorded from the subjects with different time durations [44]. The resolution of each PCG recording in the database is 16 bits, and the sampling frequency is 8 kHz. In this work, the collected PCG recordings are downsampled to 4 kHz for the time-frequency analysis. Moreover, we have also evaluated the performance of the proposed approach using the database available in 15 recorded PCG signals. These 15 PCG signals were recorded from 15 different subjects (12 males and 3 females with the age group of 27 ± 5 years) using Thinklab digital stethoscope (https://www.thinklabs.com/). The subjects have given written consent before recording the PCG signal in a noninvasive way [36]. The sampling frequency of each recorded signal is 4 kHz. In this work, we have also considered the Michigan heart sound and murmur database (MHSMD) (http://www.med.umich.edu/lrc/psb_open/html/repo/primer_heartsound/primer_heartsound.html) to evaluate the performance of the proposed method. The MHSMD contains both normal and abnormal (AS, MS, MR, and MVP) PCG signals with a sampling frequency of 44.1 kHz [63]. Each PCG signal from MHSMD has been downsampled to 4 kHz. For each database PCG recording, a Butterworth bandpass filter with a lower and upper cutoff frequency of 25 Hz and 900 Hz is used [64]. After filtering, the amplitude normalization is performed with respect to the maximum amplitude value of the PCG recording. The normalized PCG recording, *x*(*n*), is evaluated as follows [65]:

(1) $$\begin{array}{c} x\left(n\right)=\frac{x\left(n\right)}{\max\left[\left|x\left(1\right)\right|,\left|x\left(2\right)\right|,\cdots ,\left|x\left(N\right)\right|\right]}, \end{array}$$

where |*x*(*n*)|, *n* = 1, 2, ⋯, *N* is the absolute value of the amplitude of *n*th sample of the PCG recording, and *N* is the total number of samples. After normalization, the time-frequency representation of each PCG recording is computed using SCT. The following subsection describes the spline kernel-based CT for the extraction of the time-frequency matrix from PCG recording.

### 2.2. Spline Kernel-Based Chirplet Transform (SCT)

The spline kernel-based CT is the CT with a modified kernel function [51]. This modified kernel function uses different frequency rotate and frequency shift operators for the time-frequency representation of the nonstationary signal. For a PCG signal, *x*(*n*) containing *N* samples, the discrete SCT is evaluated as follows [51]:

(2) $$\begin{array}{c} T\left(\tilde{n},k\right)=\overset{N-1}{\underset{n=0}{\sum }}\bar{x}\left(n\right)w_{\sigma }\left(n-\tilde{n}\right)e^{-j\left(2\pi nk/N\right)}\text{ for }\tilde{n}\in \left(n_{i},n_{i+1}\right), \end{array}$$

with $\bar{x}\left(n\right)=x\left(n\right)\cdot \Psi^{R}\left(n,Q\right)\cdot \Psi^{S}\left(n,\tilde{n},Q\right)$. **T** represents the time-frequency matrix, where Ψ^*R*^(*n*, *Q*) and $\Psi^{S}\left(n,\tilde{n},Q\right)$ are the frequency-rotate and frequency-shift operators, respectively. The window function is given by [49, 50],

(3) $$\begin{array}{c} w_{\sigma }\left(n\right)=\frac{1}{\sqrt{2\pi }\sigma }e^{-\left(n^{2}/2\sigma^{2}\right)} \end{array}$$

**Figure 2:**
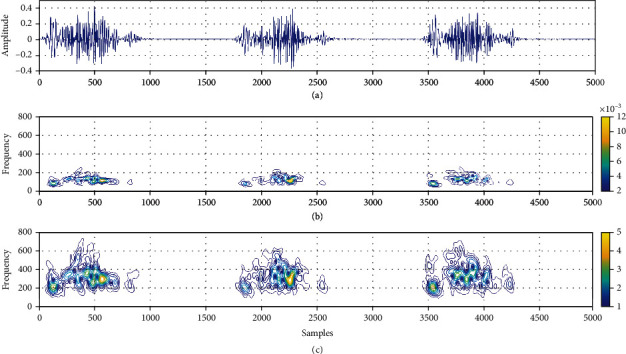
(a) Pathological PCG signal (AS pathology with murmurs present between S1-component and S2-component of each cardiac cycle). (b) Time-frequency representation of the pathological PCG signal obtained using CT. (c) Time-frequency representation of the pathological PCG signal obtained using SCT.

The frequency-rotate operator is expressed as in (4), and the frequency shift operator is as in (5) [51]:

(4) $$\begin{array}{c} \Psi^{R}\left(n,Q\right)=e^{\left(-j\overset{L}{\underset{l=1}{\sum }}q_{l}^{i}{\left(n-n_{i}\right)}^{l}+\gamma_{i}\right)}, \end{array}$$

(5) $$\begin{array}{c} \Psi^{S}\left(n,\tilde{n},Q\right)=e^{\left(j\overset{L}{\underset{l=1}{\sum }}q_{l}^{i}{\left(\tilde{n}-n_{i}\right)}^{l-1}n\right)}, \end{array}$$

where *Q*(*i*, *l*) = *q*_*l*_^*i*^ represents the local polynomial coefficient matrix for the spline kernel. The parameter *L* is denoted as the order of the spline. The parameter *γ*_*i*_ in SCT should satisfy the following conditions as [51],

(6) $$\begin{array}{c} \gamma_{i}-\gamma_{i+1}=\overset{L}{\underset{l=1}{\sum }}\frac{q_{l}^{i+1}}{l}{\left(n-n_{i}\right)}^{l}, \end{array}$$

with initial value *γ*_1_ = 0. The factor *i* = 1, 2, ⋯, *I* is the *i*th piece, where the spline is defined in a piecewise polynomial form and *I* is the total number of pieces [51]. For a pathological PCG signal, we have compared the time-frequency representations that are obtained using both CT and SCT methods. The AS pathological PCG recording is shown in Figure 2(a). The time-frequency contour plots of pathological PCG signal computed using CT and SCT are shown in Figure 2(b) and Figure 2(c), respectively. It can be observed from the figure that the time-frequency plot obtained using CT has an energy distribution between 25 Hz and 300 Hz. However, the murmurs are high-frequency sounds produced during the recording of the PCG signal [66, 67]. It is clearly observed from the time-frequency plot of the PCG recording obtained using SCT that the murmur energies are distributed between 100 Hz and 780 Hz. This shows that the information regarding the murmurs is not effectively captured in the CT-based time-frequency representation and the SCT provides better time-frequency localization for PCG recording as compared to CT.

**Figure 3:**
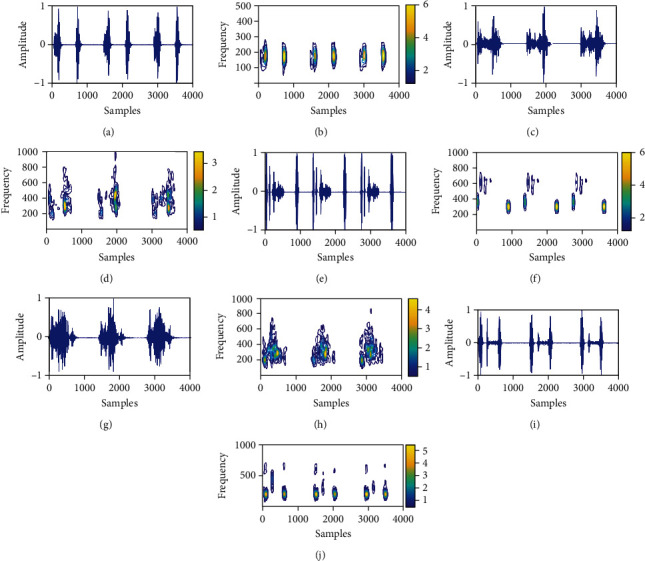
(a) Typical PCG recording of the normal class. (b) Time-frequency representation of normal PCG signal obtained using SCT. (c) PCG recording of the MR class. (d) Time-frequency representation of MR PCG signal obtained using SCT. (e) PCG recording of the MS class. (f) Time-frequency representation of MS PCG signal obtained using SCT. (g) PCG recording of the AS class. (h) Time-frequency representation of AS PCG signal obtained using SCT. (i) PCG recording of the MVP class. (j) Time-frequency representation of MVP PCG signal obtained using SCT.

The PCG signals for normal (N) and pathological classes such as MR, MS, AS, and MVP are depicted in Figure 3(a), Figure 3(c), Figure 3(e), Figure 3(g), and Figure 3(i), respectively, and the time-frequency plots for these signals were obtained using SCT are shown in Figure 3(b), Figure 3(d), Figure 3(f), Figure 3(h), and Figure 3(j), respectively. It can be observed that the pattern associated with the pathological PCG signal has different morphology for each type of HVA as compared to the normal PCG signal. The energies in the S1 and S2 components of the normal PCG signals are grossly distributed from 25 Hz to 300 Hz (as shown in Figure 3(b)). However, during HVA, the energy is distributed above 300 Hz in the time-frequency plot of the PCG signal. Each frequency component in the time-frequency matrix of the PCG recording has different characteristics for normal and pathological PCG signals. Therefore, the features computed from each frequency component of the PCG recording in the time-frequency domain will be helpful for the accurate detection of HVAs. In this study, we have extracted three types of nonlinear features, namely, L1-norm, sample entropy, and permutation entropy from the first 400 frequency atoms or components of the time-frequency representation of the PCG recording. The L1-norm (LN) features for the *k*th frequency component is evaluated as [68]

(7) $$\begin{array}{c} \text{L}{\text{N}}^{k}=\overset{N}{\underset{\tilde{n}=1}{\sum }}\left|T\left(\tilde{n},k\right)\right|. \end{array}$$

Moreover, we have also evaluated the sample entropy (SEN) [69] and permutation entropy (PEN) [70] features from the *k*th frequency atom of the matrix **T**. The features are denoted as SEN^*k*^ and PEN^*k*^. A 1200-dimensional feature vector based on the combination of 400 LN, 400 SEN, and 400 PEN features is formulated for each PCG recording obtained from the database and 15 recorded PCG signals. The KSRC classifier is used to detect HVAs from the 1200-dimensional feature vector. In the following subsection, the descriptions of DLKSRN for the classification of HVAs are presented.

### 2.3. Deep Layer KSRC

**Figure 4:**
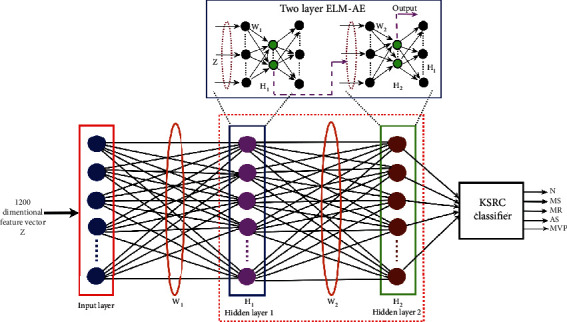
Architecture of the proposed DLKSRN for the classification of HVAs.

In this work, the DLKSRN is proposed for the classification of HVAs using PCG signal features. The architecture of DLKSRN is shown in Figure 4. It consists of an input layer, first ELM-autoencoder hidden layer, second ELM-autoencoder hidden layer, and an output layer. In this work, the hold-out and 10-fold cross-validation (CV) techniques are used to select the training and test PCG recordings. The feature matrix which comprises of the training feature vectors of the PCG recordings and the class labels are given as, {**z**_*i*_, *y*_*i*_}_*i*=1_^*m*^ with, *z*_*i*_ ∈ ℝ^*p*^ and *y*_*i*_ ∈ 1, 2, 3, 4, 5, where 1, 2, 3, 4, and 5 are class label representations for normal, MVP, AS, MR, and MS classes. *p* is the size of the feature vector obtained from each PCG recording, and *m* is the number of PCG recordings considered during the training of the DLKSRN. The hidden layer matrix in DLKSRN is evaluated by solving the following optimization problem as,

(8) $$\begin{array}{c} J=\underset{W_{i}}{\min}\left[\frac{\gamma }{2}{\left\|H_{i}W_{i}-\tilde{Z}\right\|}_{2}^{2}+{\left\|W_{i}\right\|}_{1}\right], \end{array}$$

where **W**_*i*_ is the *i*th hidden layer weight matrix and $\tilde{Z}$ is the input feature matrix for the ELM-autoencoder. For first ELM-AE, $\tilde{Z}$ is the feature matrix (**Z**) containing PCG instances and time-frequency-based features. Similarly, for the second AE, the feature matrix ($\tilde{Z}$) is the hidden layer matrix (**H**_1_) obtained from the first ELM-autoencoder. The weight matrix evaluation for each ELM-autoencoder is given by,

(9) $$\begin{array}{c} W_{i}={\left(H_{i}^{\text{T}}H_{i}+\frac{I}{\gamma }\right)}^{-1}H_{i}^{\text{T}}\tilde{Z}. \end{array}$$

The feature matrix obtained in the second hidden layer of ELM-autoencoder is given as follows:

(10) $$\begin{array}{c} Z^{*}=f\left(f\left(W_{1}Z\right)W_{2}\right). \end{array}$$

The new feature matrix, **Z**^∗^, is used as the input to the KSRC layer of the proposed DLKSRN for the classification. KSRC is a kernel-based sparse representation technique, and it does not require rigorous training like deep neural networks (DNNs) to evaluate the class labels of the test feature vectors [60, 61]. It consists of four steps to estimate the class label of test PCG feature vectors. These steps are (i) mapping of the feature vectors of PCG signal into higher dimension space using kernel function, (ii) use of kernel-based dimension reduction for feature reduction, (iii) evaluation of coefficient vector and residual to test PCG feature vector by solving L1-norm optimization problem, and (iv) assignment of the class label to test PCG vector based on finding the minimum distance for all classes [60, 61]. The SRC has less performance when the feature vectors are not linearly separable. To overcome this limitation, KSRC maps the input PCG feature vector to a higher dimension space and performs the SRC in that new space.

The mapping function Ψ(**z**^∗^) projects each training feature vector to a higher dimensional space, and it is given as Ψ(**z**^∗^) = [Ψ_1_(**z**^∗^), Ψ_2_(**z**^∗^), ⋯⋯⋯Ψ_*r*_(**z**^∗^)]^*T*^, where Ψ(**z**^∗^) ∈ ℝ^*r*^ with *r* ≫ *p* is the dimension of new feature space or higher-dimension space. In new feature space, we can represent the mapped feature vector, Ψ(**z**_*t*_^∗^), as the linear combination of the mapped training feature vectors of the PCG recordings, and it is given by Ψ(**z**_*t*_^∗^) = ∑_*i*=1_^*p*^*γ*_*i*_Ψ(**z**_*i*_^∗^) = Ψ*γ*, where *γ* = [*γ*_1_, *γ*_2_,. ⋯ ⋯⋯⋯*γ*_*m*_]^*T*^ is the coefficient vector, and it can be evaluated based on the solution of the following optimization problem as [60, 61]

(11) $$\begin{array}{c} \underset{\gamma }{\min}{\left\|\gamma \right\|}_{1,} \end{array}$$

subjected to Ψ(**z**_*t*_^∗^) = Ψ*γ*. In KSRC, the dimension of kernel space *r* is higher as compared to the second hidden layer space $\tilde{p}$, and also, it can be higher than the number of training instances *m*. Therefore, for getting a sparse solution of *γ* in (11), the dimension reduction step is used in the kernel space. The dimension reduction is performed based on the use of the transformation matrix **A**. The constraint for the optimization problem in (11) is modified as follows:

(12) $$\begin{array}{c} A^{T}\Psi \left(z_{t}^{*}\right)=A^{T}\Psi \gamma , \end{array}$$

where the transformation matrix can be evaluated as follows:

(13) $$\begin{array}{c} A=\Psi S. \end{array}$$

The matrix **S** is the pseudotransformation matrix, and it is evaluated using any one of the dimension reduction techniques (random projection, kernel principal component analysis (KPCA), and kernel linear discriminant analysis (KLDA)) [60, 61]. The expression in (12) can be simplified as follows:

(14) $$\begin{array}{c} \Psi^{T}\Psi \left(z_{t}^{*}\right)S^{T}=\Psi^{T}\Psi \gamma S. \end{array}$$

The above equation can also be written as, **S**^*T*^*k*(**z**_*t*_^∗^, **z**_*i*_^∗^) = **K***γ ***S**, where *k*(**z**_*t*_^∗^, **z**_*i*_^∗^) and **K** are the kernel function and the kernel matrix, respectively. The original optimization problem in KSRC is modified as follows:

(15) $$\begin{array}{c} \underset{\gamma }{\min}{\left\|\gamma \right\|}_{1}, \end{array}$$

subject to **S**^*T*^*k*(**z**_*i*_^∗^, **z**_*t*_) = **S**^*T*^**K***γ*. The residual for the test instance **z**_*t*_^∗^ for the *c*th class is obtained as follows [60]:

(16) $$\begin{array}{c} \text{r}{\text{s}}_{c}\left(z_{t}^{*}\right)={\left\|S^{T}k\left(z_{t}^{*},z_{i}^{*}\right)-S^{T}K\delta_{c}\right\|}_{2}, \end{array}$$

where *δ*_*c*_ = [*δ*_*c*_(*γ*_1_), *δ*_*c*_(*γ*_2_), ⋯, *δ*_*c*_(*γ*_*m*_)], and *δ*_*c*_(*γ*_*i*_) is the characteristic function for the *c*th class. This function is defined as follows [60, 61]:

(17) $$\begin{array}{c} \delta_{c}\left(\gamma_{i}\right)=\left\{\begin{array}{cc} \gamma_{i} & \text{if }y_{i}=c\\ 0 & \text{elsewhere} \end{array}\right\}. \end{array}$$

The residual for each class is computed, and the final class label for the second hidden layer feature vector of test PCG recording is given by

(18) $$\begin{array}{c} y_{t}=\arg\underset{c=1,2..\cdots C}{\min}\text{r}{\text{s}}_{c}\left(z_{t}^{*}\right). \end{array}$$

In this study, the number of neurons used in the first and second hidden layers of the proposed DLKSRN is 800 and 600, respectively. Moreover, we have also considered the random forest (RF) [36] and *K*-nearest neighbour (KNN) [65] classifiers for the classification of HVAs from the feature vectors of test PCG recordings. The optimal parameters of the RF classifier [71] such as the number of trees, number of splits for each decision tree, and depth of each decision tree obtained using the grid-search technique are 20, 20, and 15, respectively. For the KNN classifier, we have considered the number of the nearest neighbours as 3 and used Euclidean as the distance metric [72]. The performance of the 1200 dimensional SCT-based time-frequency features of PCG recordings is evaluated using DLKSRN, KSRC, RF, and KNN classifiers with the hold-out cross-validation (CV) strategy. For hold-out CV, 60%, 10%, and 30% of PCG signal instances are considered for training, validation, and testing of the DLKSRN classifier. Similarly, for the 10-fold CV case, 90% of PCG signal instances from the feature matrix are used to train the DLKSRN classifier. The remaining 10% PCG signal instances are evaluated during the testing phase of the DLKSRN classifier in each fold. The metrics, namely, the sensitivity, specificity, precision, *F*-score, and overall accuracy (OA), are used to evaluate the performance of DLKSRN, KSRC, RF, and KNN classifiers [72]. In the following section, the results obtained using the proposed approach are discussed in detail.

## 3. Results and Discussion

**Figure 5:**
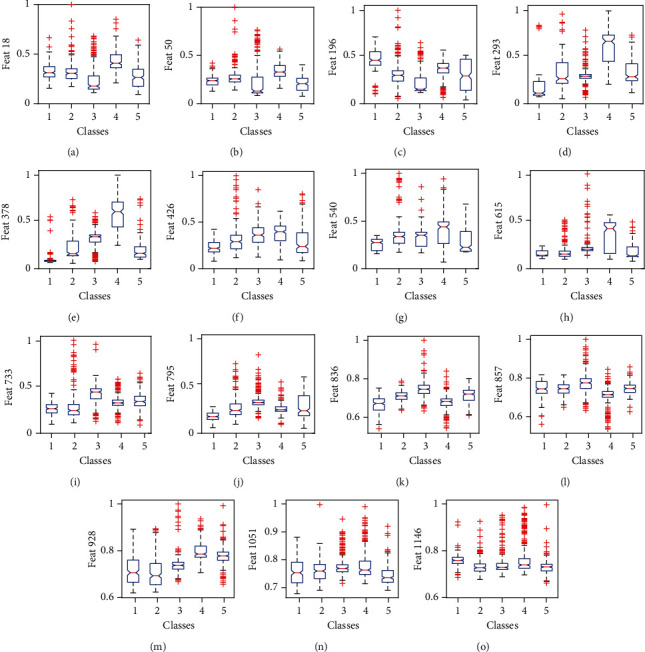
(a) Boxplot of 18th L1 norm feature or attribute (Feat 18) for all classes. (b) Boxplot of 50th L1 norm attribute (Feat 50) for all classes. (c) Boxplot of 196th L1 norm attribute (Feat 196) for all classes. (d) Boxplot of the 293rd L1 norm attribute (Feat 293) for all classes. (e) Boxplot of the 378th L1 norm attribute (Feat 378) for all classes. (f) Boxplot of the 26th SENT attribute (Feat 26) for all classes. (g) Boxplot of 140th SENT attribute (Feat 140) for all classes. (h) Boxplot of the 215th SENT attribute (Feat 215) for all classes. (i) Boxplot of the 333th SENT attribute (Feat 333) for all classes. (j) Boxplot of the 395th SENT attribute (Feat 395) for all classes. (k) Boxplot of the 36th PENT attribute (Feat 36) for all classes. (l) Boxplot of the 57th PENT attribute (Feat 57) for all classes. (m) Boxplot of the 128th PENT attribute (Feat 128) for all classes. (n) Boxplot of the 251st PENT attribute (Feat 251) for all classes. (o) Boxplot of the 346th PENT attribute (Feat 346) for all classes.

In the first part of this section, the statistical analysis results of SCT-based features of PCG recordings are presented. In the second part, the classification results using RF, KNN, KSRC, and the proposed DLKSRN models are shown. The third part of this section describes the comparison and advantages of the proposed approach for HVA detection. In this study, we have conducted a statistical analysis of all 1200 SCT-based features of the PCG recording. The results are shown for 15 different features out of 1200 features. The intraclass variations of the LN features for 18th, 50th, 196th, 293th, and 378th frequency components for all N, MVP, AS, MR, and MS categories are depicted in Figures 5(a)–5(e), respectively. Similarly, the within-class variations of the SEN features for the 26th, 140th, 250th, 333th, and 395th frequency components for all classes are shown in Figures 5(f)–5(j), respectively. Moreover, in Figures 5(k)–5(o), we have shown the intraclass variations of PEN features for 36th, 57th, 128th, 251st, and 346th frequency components in all classes. The parameters such as mean and standard deviation values of features whose intraclass variations given in boxplots of Figure 3 are shown in Table 1. It is noted that each feature has distinct mean values for each of the pathological classes (AS, MS, MR, MVP) and normal class. The SEN feature for more than 300 frequency components of the SCT-based time-frequency matrix has a lower mean value for the normal class as compared to the pathological classes. Similarly, more than 230 PEN features have lower mean values for the normal class, and more than 200 L1-norm features have higher mean values for the AS class. The pathological signature for MS is the presence of diastolic murmurs [73], and murmurs are observed between the systolic interval of PCG recording in MVP pathology [74]. In MS and AS pathologies, the murmurs have low-pitch sounds. Similarly, the high-pitch sounds are observed in the PCG recording during AR-based HVA [14]. The aforementioned pathological changes on the PCG recording affect the morphologies of the SCT-based time-frequency matrices. Hence, the features from the time-frequency matrices have distinct mean and standard deviation values. We have also used the analysis of variance- (ANOVA-) based test [75] to verify the statistical significance of SCT-based time-frequency features. It is observed from the ANOVA test that all 1200 features extracted from the SCT-based time-frequency representation of PCG recording have *p* values less than 0.001 and is significant for the detection of HVAs.

**Figure 6:**
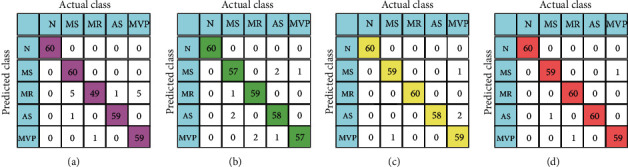
Confusion matrix of classifier obtained using 1200 dimensional feature vectors with PCG signals: (a) RF classifier; (b) KNN classifier; (c) KSRC; and (d) DLKSRN.

**Figure 7:**
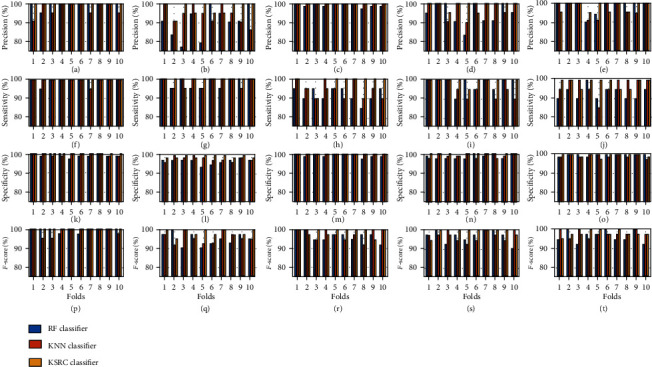
Precision values of the KSRC, RF, and KNN classifiers obtained using the 10th fold cross-validation for (a) normal class, (b) MS class, (c) MR class, (d) AS class, and (e) MVP class. Sensitivity values of the KSRC, RF, and KNN classifiers obtained using the 10th fold cross-validation for (f) normal class, (g) MS class, (h) MR class, (i) AS class, and (j) MVP class. Specificity values of the KSRC, RF, and KNN classifiers obtained using the 10th fold cross-validation for (k) normal class, (l) MS class, (m) MR class, (n) AS class, and (o) MVP class. *F*-score values of the KSRC, RF, and KNN classifiers obtained using the 10th fold cross-validation for (p) normal class, (q) MS class, (r) MR class, (s) AS class, and (t) MVP class.

The classification results of RF, KNN, KSRC, and DLKSRN are shown in Table 2. In this work, we have considered five random trials based on the hold-out CV to evaluate the performance of each classifier. The performance metrics are shown in the mean and standard deviation format in Table 2. Also, we have shown the confusion matrices of RF, KNN, KSRC, and DLKSRN classification models in Figures 6(a)–6(d), respectively. It is evident that the average number of true positives is high using DLKSRN as compared to the RF, KNN, and KSRC classifiers. The number of true positive (TP), true negative (TN), false positive (FP), and false-negative (FN) values is listed in Table 2 for normal and other pathological classes. It can be observed that DLKSRN has less number of average FN and FP values for each class. The values of the metrics such as precision, sensitivity, specificity, and *F*-score are also high using DLKSRN as compared to other classification approaches. It is also observed that the average accuracy of DLKSRN is higher than KSRC, RF, and KNN classifiers. The DLKSRN classification results for 10-fold CV are shown in Table 3. The classification results of KSRC, RF, and KNN classifiers with 10-fold CV are depicted in Figure 7. The precision values for normal, MS, MR, AS, and MVP classes over all 10 folds are depicted in Figures 7(a)–7(e). Similarly, the sensitivity values for all classes with ten-fold CV are shown in Figures 7(f)–7(j). In Figures 7(k)–(o), we have shown the variation in specificity values for all ten folds for all classes. Moreover, the *F*-score values are depicted in Figures 7(p)–7(t) for normal, MS, MR, AS, and MVP classes. It is observed from the results that for MS class, the sensitivity is 95% after three folds and reached 100% for other folds. Similarly, the specificity values of DLKSRN for the first, second, fourth, eighth, and tenth fold are more than 98.5%. Similarly, for the MR class, the sensitivity values of KSRC for the second, third, and fourth folds are less than the remaining folds. For the MR class, the specificity values for all folds of both DLKSRN and KSRC are 100%. Similar variations are also observed for other pathological cases using the DLKSRN classifier. It is also observed that the sensitivity, specificity, precision, and *F*-score values of DLKSRN are higher for all folds as compared to RF classifiers for each class. In some folds, the KNN and KSRC have demonstrated similar sensitivity, specificity, and *F*-score values. The overall accuracy of DLKSRN for 10-fold CV using SCT-based time-frequency features is 99.24%. This value is higher than the average accuracy values of KSRC (98.90%), KNN (97.12%), and RF (96.12%) classifiers. It can be noted that the nonlinear features extracted using SCT of PCG recording are able to classify the HVAs accurately using DLKSRN. In this study, the parameters of the DLKSRN classifier such as the number of neurons in the 1st and 2nd hidden layers are selected based on the maximum accuracy values in the validation and test sets. The variations in the overall accuracy values with hidden neurons in the 1st and 2nd hidden layers are shown in Table 4. It can be observed from the table that the overall accuracy value of the DLKSRN classifier is high when the number of neurons in the 1st and 2nd hidden layers are 800 and 600, respectively. The overall accuracy value decreases by increasing the number of neurons in both hidden layers. Similarly, for the MHSMD database, the classification results obtained using the DLKSRN classifier are shown in Table 5. It is observed that the proposed SCT-based time-frequency domain features combined with the DLKSRN classifier have obtained an overall accuracy value of 96.79%. The sensitivity and specificity values are greater than 94% for each class using the DLKSRN classifier. Moreover, we have tested the effectiveness of our proposed approach with 15 recorded PCG signals. The DLKSRN model which has been trained using the features from the public database has been used to test the performance of the private database. The LN, SEN, and PEN features are extracted from all 15 recorded PCG signals. The trained DLKSRN model successfully predicted all 15 feature vectors of PCG recordings as normal class thereby showing the effectiveness of the proposed approach for real-time precision of HVAs.

The objective of this study is the HVA detection using nonlinear features extracted from the SCT-based time-frequency analysis of PCG recording. The proposed features are found to be discriminative with the lowest *p* values obtained using the statistical test. The classification results obtained using the hold-out and 10-fold CV-based PCG instance selection reveal that the proposed approach has obtained an overall accuracy of more than 99% for the detection of HVA. A comparison with the existing algorithms for automated HVA detection is shown in Table 6. Safara et al. [76] developed the automated approach using wavelet packet decomposition-based feature extraction technique and SVM classifier for the discrimination of MR, AS, and AR-based HVAs with PCG recordings. They have achieved an accuracy of 97.56%. Maglogiannis et al. [77] used the SVM classifier coupled with the morphological features (standard values of S1 and S2 peaks and other features) for the detection of MR and MS pathologies and reported an accuracy of 91.23% in classifying two HVAs. Moreover, Zheng et al. [78] employed the energy fraction and energy-based features coupled with the SVM classifier for the automated discrimination of HVAs such as tricuspid insufficiency (TI), pulmonary stenosis (PS), mitral insufficiency (MI), and AS. They have obtained an overall accuracy of 97.17% in classifying the four HVAs. The time-frequency domain magnitude and phase features extracted using the SST of PCG signal have been used in [36] for the discrimination of AS, MS, and MR classes. They have obtained an overall accuracy value of 95.12% in classifying the three classes. The combination of MFCC- and DWT-based features extracted PCG signal, along with SVM classifier, has been used for automated HVA detection with an overall accuracy of 97.9% [44]. The CT-based time-frequency features obtained from PCG and composite classification model yielded an overall accuracy value of 98.33% [13]. Oh et al. [79] have proposed a waveNet-based DNN model for the classification of HVAs using PCG recordings and obtained an overall accuracy value of 98.20%. The proposed approach demonstrated higher classification performance as compared to the existing algorithms for automated HVA detection. The method reported in [13] has classified AS, MS, and MR pathologies using PCG. However, in the present work, we have considered MVP pathology along with AS, MS, and MR ailments for the development of an automated HVA detection system. The advantages of the proposed HVA detection approach are given as follows:

- The SCT has demonstrated better time-frequency localization for both normal and pathological PCG signals as compared to CT
- The proposed approach used the nonlinear features from different frequency components of SCT-based time-frequency representation of the PCG signal
- The DLKSRN based on the ELM-autoencoder and KSRC is proposed for the classification of HVAs
- The proposed approach is tested using the recorded PCG signals

In this work, the local features from the frequency components of the time-frequency representation of the PCG signal are evaluated. The two-dimensional convolutional autoencoder [80] can be used for the extraction of learnable features from the SCT-based time-frequency representation of the PCG signal for the classification of HVAs. The sparse residual entropy features [81] and wavelet bispectrum-based features [82] can be used for the detection of HVAs from the PCG signal. The convolutional neural network [83], convolutional attention-based network [84], and other deep learning methodologies [85] can be used for the detection of HVAs without using extracted features from PCG recordings.

## 4. Conclusion

A novel HVA detection approach using PCG signals is proposed. This approach used SCT to compute the time-frequency representation of PCG recording. The nonlinear features (LN, SEN, and PEN) are computed from the frequency components of time-frequency representation. The DLKSRN classifier is used to discriminate automatically into four categories of HVA classes using the extracted features. The proposed approach demonstrated an average accuracy of 99.23% and 99.24% using hold-out and 10-fold CV methods. The proposed approach is also evaluated using the recorded signal, and the result obtained shows the practicality of the proposed approach. In the future, we intend to extend this method to detect coronary artery disease and psychological stress using PCG signals. The approach can also be implemented in real-time for IoMT applications.

## Figures and Tables

**Table 1:** Mean ± SD of the selected features extracted from the time-frequency matrix for the five pathological classes.

Feature	Feature number	N	MS	MR	AS	MVP
L1-norm	Feat 18	0.3212 ± 0.0796	0.3231 ± 0.1094	0.2446 ± 0.1478	0.4286 ± 0.1009	0.2699 ± 0.1105
	Feat 50	0.2374 ± 0.0530	0.2802 ± 0.1110	0.2179 ± 0.1701	0.3402 ± 0.0793	0.2086 ± 0.0789
	Feat 196	0.4748 ± 0.1212	0.3154 ± 0.1460	0.2217 ± 0.1392	0.3597 ± 0.1128	0.2995 ± 0.1706
	Feat 293	0.1498 ± 0.1256	0.3104 ± 0.1690	0.2774 ± 0.1182	0.6083 ± 0.2066	0.3155 ± 0.1425
	Feat 378	0.0525 ± 0.0637	0.1898 ± 0.1380	0.2782 ± 0.1184	0.5697 ± 0.1914	0.1652 ± 0.1186
SEN	Feat 426	0.2315 ± 0.0693	0.3213 ± 0.1580	0.3747 ± 0.1238	0.3820 ± 0.1034	0.2906 ± 0.1501
	Feat 540	0.2467 ± 0.0591	0.3490 ± 0.1531	0.3242 ± 0.0965	0.3919 ± 0.1787	0.2908 ± 0.1318
	Feat 615	0.1600 ± 0.0325	0.1864 ± 0.0908	0.2342 ± 0.1238	0.3418 ± 0.1588	0.1852 ± 0.0766
	Feat 733	0.2613 ± 0.0724	0.3046 ± 0.1837	0.4197 ± 0.1099	0.3354 ± 0.0858	0.3437 ± 0.0961
	Feat 795	0.2175 ± 0.0536	0.3343 ± 0.1465	0.4140 ± 0.1088	0.3162 ± 0.0715	0.3490 ± 0.1617
PEN	Feat 836	0.6661 ± 0.0408	0.7094 ± 0.0279	0.7490 ± 0.0495	0.6783 ± 0.0431	0.7142 ± 0.0390
	Feat 857	0.7657 ± 0.0378	0.7635 ± 0.0276	0.7927 ± 0.0521	0.7302 ± 0.0429	0.7658 ± 0.0285
	Feat 928	0.7174 ± 0.0613	0.7040 ± 0.0595	0.7429 ± 0.0480	0.7964 ± 0.0401	0.7751 ± 0.0431
	Feat 1051	0.7543 ± 0.0430	0.7628 ± 0.0387	0.7815 ± 0.0417	0.7849 ± 0.0553	0.7469 ± 0.0385
	Feat 1146	0.7610 ± 0.0270	0.7363 ± 0.0368	0.7490 ± 0.0550	0.7675 ± 0.0692	0.7319 ± 0.0353

**Table 2:** Classification results obtained for automated detection of HVAs using various classifiers with SCT domain features and hold-out CV.

Classifier	Class	Performance measure								OA (%)
		TP	TN	FP	FN	Precision (%)	Sensitivity (%)	Specificity (%)	*F*-score (%)	
RF	N	60	227	0	0	97.75 ± 2.63	98.33 ± 2.35	99.38 ± 0.74	98.00 ± 1.50	95.66
	MS	60	227	6	0	88.60 ± 3.77	96.99 ± 1.39	96.72 ± 1.30	92.56 ± 1.90	
	MR	49	238	1	11	96.31 ± 1.87	87.99 ± 4.62	99.13 ± 0.42	91.94 ± 3.20	
	AS	59	228	1	1	92.38 ± 3.59	95.33 ± 1.82	97.90 ± 1.04	93.79 ± 1.63	
	MVP	59	228	5	1	98.22 ± 1.24	93.32 ± 4.56	99.56 ± 0.31	95.67 ± 2.79	
KNN	N	60	231	0	0	98.69 ± 1.35	99.66 ± 0.74	99.64 ± 0.37	99.17 ± 0.82	97.00
	MS	57	234	3	3	90.16 ± 1.65	97.66 ± 0.91	97.28 ± 0.49	93.76 ± 1.28	
	MR	59	232	2	1	97.91 ± 1.46	93.99 ± 3.45	99.48 ± 0.35	95.89 ± 2.19	
	AS	58	233	3	2	98.24 ± 2.11	91.99 ± 3.20	99.57 ± 0.52	94.99 ± 2.20	
	MVP	57	234	1	3	96.41 ± 2.56	97.33 ± 1.90	99.04 ± 0.71	96.85 ± 1.76	
KSRC	N	60	236	0	0	100.0 ± 0.00	100.0 ± 0.00	100.0 ± 0.00	100.0 ± 0.00	98.66
	MS	59	237	1	1	96.13 ± 2.12	98.99 ± 0.91	98.98 ± 0.56	97.54 ± 1.52	
	MR	60	236	0	0	98.66 ± 2.17	97.66 ± 1.90	99.66 ± 0.55	98.15 ± 1.89	
	AS	58	238	0	2	99.00 ± 1.49	96.99 ± 2.47	99.74 ± 0.38	97.96 ± 1.29	
	MVP	59	237	3	0	96.70 ± 1.99	96.66 ± 1.17	99.15 ± 0.51	96.66 ± 1.01	
DLKSRN	N	60	238	0	0	100.0 ± 0.00	100.0 ± 0.00	100.0 ± 0.00	100.0 ± 0.00	99.23
	MS	59	239	1	1	98.24 ± 1.02	99.01 ± 0.10	99.00 ± 0.63	99.13 ± 0.14	
	MR	60	238	1	0	99.02 ± 0.23	98.76 ± 1.25	99.88 ± 0.16	99.00 ± 0.26	
	AS	60	238	0	1	99.18 ± 0.64	97.95 ± 1.38	99.82 ± 0.01	98.72 ± 1.33	
	MVP	59	239	1	1	96.89 ± 1.02	97.36 ± 1.29	99.56 ± 0.11	97.51 ± 1.82	

**Table 3:** Results of the classification using the DLKSRN classifier with ten-fold CV.

HVDs	Measures (%)	Fold 1	Fold 2	Fold 3	Fold 4	Fold 5	Fold 6	Fold 7	Fold 8	Fold 9	Fold 10	Average
N	Precision	100.0	100.0	100.0	100.0	100.0	100.0	100.0	100.0	100.0	100.0	100.0 ± 0.00
	Sensitivity	100.0	100.0	100.0	100.0	100.0	100.0	100.0	100.0	100.0	100.0	100.0 ± 0.00
	Specificity	100.0	100.0	100.0	100.0	100.0	100.0	100.0	100.0	100.0	100.0	100.0 ± 0.00
	*F*-score	100.0	100.0	100.0	100.0	100.0	100.0	100.0	100.0	100.0	100.0	100.0 ± 0.00
MS	Precision	100.0	90.90	100.0	95.23	100.0	95.23	95.23	100.0	100.0	100.0	97.65 ± 3.27
	Sensitivity	100.0	100.0	95.00	100.0	100.0	100.0	100.0	100.0	100.0	100.0	99.50 ± 1.58
	Specificity	98.75	98.75	100.0	98.73	100.0	100.0	100.0	98.75	100.0	98.73	99.37 ± 0.66
	*F*-score	100.0	95.23	100.0	97.56	100.0	97.56	100.0	97.43	97.56	100.0	98.53 ± 1.69
MR	Precision	100.0	100.0	100.0	100.0	100.0	100.0	100.0	100.0	100.0	100.0	100.0 ± 0.00
	Sensitivity	100.0	95.00	90.00	95.00	100.0	100.0	100.0	100.0	100.0	100.0	98.00 ± 3.49
	Specificity	100.0	100.0	100.0	100.0	100.0	100.0	100.0	100.0	100.0	100.0	100.0 ± 0.00
	*F*-score	100.0	97.43	100.0	97.43	100.0	100.0	100.0	100.0	94.73	100.0	98.95 ± 1.83
AS	Precision	100.0	100.0	95.23	100.0	100.0	95.00	100.0	100.0	100.0	100.0	99.02 ± 2.06
	Sensitivity	100.0	100.0	100.0	100.0	95.00	100.0	100.0	100.0	95.00	100.0	99.00 ± 2.10
	Specificity	100.0	100.0	100.0	98.73	100.0	100.0	100.0	97.46	100.0	100.0	98.98 ± 1.80
	*F*-score	94.73	100.0	97.56	100.0	100.0	100.0	100.0	100.0	100.0	97.56	98.98 ± 1.80
MVP	Precision	95.23	100.0	100.0	95.00	100.0	95.23	100.0	95.23	100.0	100.0	98.06 ± 2.49
	Sensitivity	100.0	100.0	95.00	100.0	100.0	100.0	95.00	95.00	100.0	100.0	98.50 ± 2.41
	Specificity	100.0	100.0	98.75	100.0	97.50	100.0	100.0	100.0	100.0	98.73	98.00 ± 1.54
	*F*-score	95.23	97.43	97.43	100.0	100.0	97.43	100.0	97.56	97.56	97.43	98.00 ± 1.54
All	OA	100.0	99.33	99.04	100.0	100.0	98.05	98.11	99.25	99.86	98.84	99.24 ± 0.74

**Table 4:** Overall accuracy values obtained using the DLKSRN classifier for various number of neurons in the 1st and 2nd hidden layer of validation and test sets for N vs. MS vs. MR vs. AS vs. MVP classification scheme.

Number of neurons		Overall accuracy (%)	
1^st^ hidden layer	2^nd^ hidden layer	Validation set	Test set
200	100	94.60	95.86
400	200	96.00	96.26
600	400	96.20	96.66
800	600	97.80	99.23
1000	800	95.80	97.33

**Table 5:** Classification results obtained for automated detection of HVAs using the DLKSRN classifiers with SCT domain features and hold-out CV.

Database used	Class	Precision (%)	Sensitivity (%)	Specificity (%)	*F*-score (%)	OA (%)
MHSMD	N	98.07 ± 2.07	100 ± 0.00	99.48 ± 0.56	99.01 ± 1.06	96.79 ± 0.18
	MS	93.87 ± 2.98	95.99 ± 0.90	98.38 ± 0.80	94.90 ± 1.73	
	MR	96.42 ± 2.35	96.66 ± 2.36	99.05 ± 0.63	96.50 ± 0.69	
	AS	98.30 ± 1.68	94.33 ± 1.90	99.57 ± 0.43	96.25 ± 0.77	
	MVP	97.23 ± 2.29	96.33 ± 2.17	99.22 ± 0.56	96.64 ± 1.01	

**Table 6:** Summary of automated detection of HVA developed using PCG signals using the same database.

Methods used for feature extraction	Classifiers	Classes	Accuracy (%)
Morphological features extracted from PCG recording [77]	SVM	N, MS, MR	91.23
Wavelet entropies as features from PCG [86]	ANFIS	N, PS, MS	98.33
Multilevel basis selection- (MLBS-) based wavelet features extracted from PCG [76]	SVM	N, AS, MR, AR	97.56
Entropy and energy fraction-based features [78]	SVM	N, TI, PS, MI, MS	97.17
Wavelet and MFCC features obtained from PCG [44]	SVM	N, AS, MS, MR, MVP	97.90
Magnitude and phase features extracted using SST of PCG [36]	Random forest	N, AS, MS, MR	95.13
Features extracted using CT of PCG [13]	Multiclass composite classifier	HC, AS, MS, MR	98.33
DNN [79]	WaveNet	N, MS, MR, AS, MVP	98.20
Proposed work (features evaluated in SCT domain of PCG)	DLKSRN	N, MS, MR, AS, MVP	99.24

## Data Availability

The codes and the classification results of the proposed work are available upon request to the authors.
